# Dual approach for large mediastinal tumors in the thoracic outlet: transmanubrial osteomuscular sparing approach and video-assisted thoracoscopic surgery

**DOI:** 10.1186/s13019-019-0863-5

**Published:** 2019-02-26

**Authors:** Hidenao Kayawake, Toyofumi F. Chen-Yoshikawa, Hiroshi Date

**Affiliations:** Department of Thoracic Surgery, Kyoto University, 54 Shogoin-Kawahara-cho, Sakyo-ku, Kyoto, 606-8507 Japan

**Keywords:** Thoracic outlet tumor, Transmanubrial osteomuscular sparing approach, Dual approach

## Abstract

**Background:**

Selecting the proper surgical approach for mediastinal tumors in the thoracic outlet is difficult. Video-assisted thoracoscopic surgery is ideal because of the less invasiveness; however, it is often difficult to resect tumors only by video-assisted thoracoscopic surgery due to the poor visualization of the cranial side of tumors. We report two successfully treated cases by using a dual approach consisting of the transmanubrial osteomuscular sparing technique and video-assisted thoracoscopic surgery for aiming both the less invasiveness and the good visualization of the cranial side of tumors.

**Case presentations:**

We present two resected cases of the mediastinal tumor in the thoracic outlet. The first case was a 28-year-old woman and the second case was a 37-year-old man. They had a mediastinal tumor in the thoracic outlet which was detected on the roentgenogram. A definitive preoperative diagnosis was unavailable. The surgical resection was started with video-assisted thoracoscopic surgery in the both cases. After the dissection of the caudal side of the tumor, the dissection of the cranial side was judged to be difficult and risky because the tumor was located adjacent to major vessels and the good visualization of this side couldn’t be acquired. Therefore, the transmanubrial approach was sequentially performed and complete resection was safely achieved. Postoperatively, although transient Horner syndrome appeared in both cases, they recovered from this syndrome and were discharged. The final diagnosis was schwannoma for both cases. Neither of the cases had any functional restriction of the upper extremity.

**Conclusions:**

This dual approach for mediastinal tumors in the thoracic outlet is useful in terms of safety and lower invasiveness.

## Background

Selecting the appropriate surgical approach for mediastinal tumors in the thoracic outlet is sometimes difficult owing to anatomical issues. A minimally invasive surgical approach such as video-assisted thoracoscopic surgery (VATS) is ideal. However, for large tumors that are adjacent to major vessels, dissection of the cranial side is sometimes difficult with VATS; therefore, an additional surgical approach by which the good visualization of this side is obtained can be required for safety. For this purpose, the transmanubrial osteomuscular sparing approach (TMA) is useful because it allows good visualization of the cervical major vessels such as subclavian veins and arteries. We report two cases of mediastinal tumors in the thoracic outlet that were successfully treated with the dual surgical approach of TMA and VATS.

## Case presentations

### Case 1

**Fig. 1:**
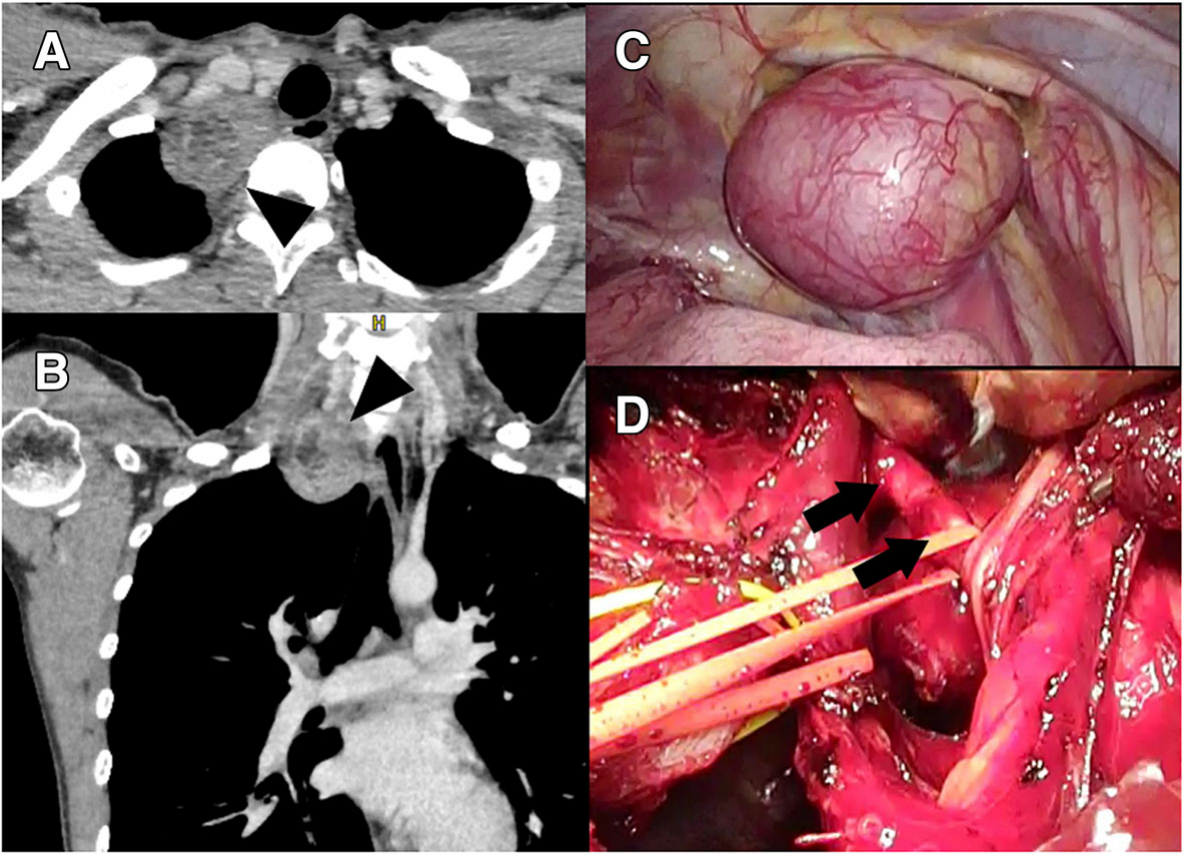
Axial (**a**) and coronal (**b**) views of the chest computed tomogram. The tumor (arrowhead) was in the thoracic outlet and adjacent to the right brachiocephalic artery and right subclavian vein. Intraoperative findings with video-assisted thoracoscopic surgery (VATS) (**c**) and the transmanubrial osteomuscular sparing approach (TMA) (**d**). The tumor was safely resected through the dual approach of VATS and TMA after encircling the right brachiocephalic artery (arrow)

A 28-year-old woman was admitted for the surgical treatment of a mediastinal tumor that was detected on a chest roentgenogram. Chest computed tomography (CT) showed that her mediastinal tumor was in the right thoracic outlet and adjacent to the right subclavian vein and right brachiocephalic artery (Fig. 1a, b). A definitive preoperative diagnosis was unavailable. The tumor was suspected to be a benign neurinoma with a possibility of malignancy. The operation was started with VATS, in preparation for TMA. The patient was placed in the left semi-lateral decubitus position. Three access ports were placed at the 5th intercostal space on the middle axillary line, the 3rd intercostal space on the middle axillary line, and the 5th intercostal space on the anterior axillary line. Dissection of the caudal side could be performed with VATS (Fig. 1c); however, safe dissection of the cranial side was difficult and risky because of the low mobility of the tumor, poor visualization, difficulty in handling surgical devices and tumor location (adjacent to right subclavian vein and right brachiocephalic artery). Therefore, TMA was sequentially performed. Because TMA allows good visualization of the cervical vessels and nerves, the cranial side was safely dissected, and the tumor was completely resected (Fig. 1d). The intraoperative diagnosis was a benign tumor compatible with a neurogenic tumor. Postoperatively, Horner syndrome was detected transiently and resolved naturally. The patient was discharged 6 days postoperatively. The final diagnosis was schwannoma, and the tumor was completely resected. Three months after the operation, she was free of Horner syndrome and any functional restriction of the right upper extremity.

### Case 2

**Fig. 2:**
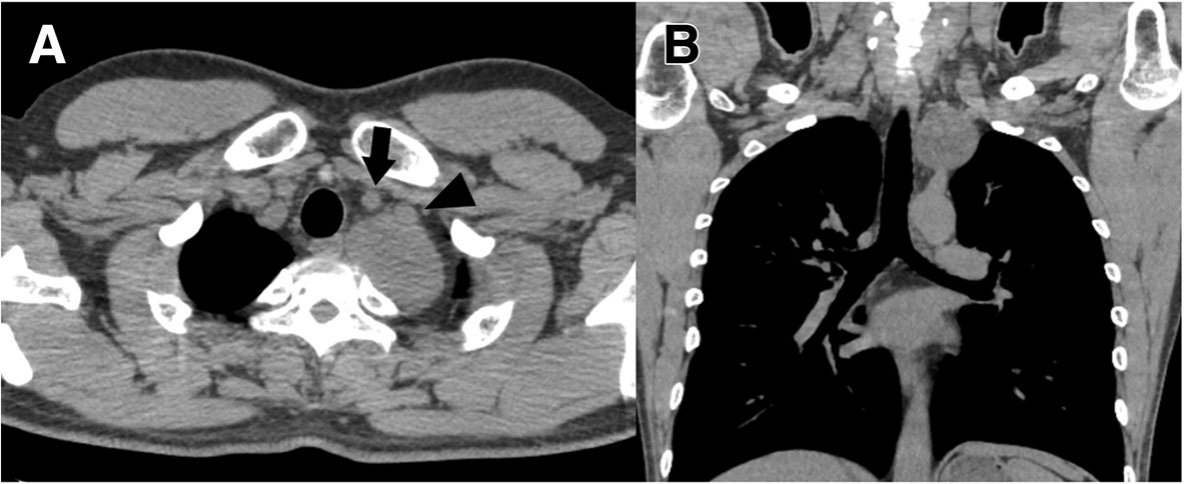
Axial (**a**) and coronal (**b**) views of the chest computed tomogram. The tumor was adjacent to the left common carotid (arrow) and left subclavian (arrowhead) arteries

A 37-year-old man was admitted for the surgical treatment of a mediastinal tumor that was detected on a chest roentgenogram. CT showed that his mediastinal tumor was in the left thoracic outlet and adjacent to the left common carotid and left subclavian arteries (Fig. 2). A definitive preoperative diagnosis was unavailable. The operation was started with VATS, in preparation for TMA. The patient was placed in the right semi-lateral decubitus position. Three access ports were placed at the 5th intercostal space on the anterior axillary line, the 3rd intercostal space on the midclavicular line, and the 3rd intercostal space on the anterior axillary line. Dissection of the caudal side was performed with VATS; however, the cranial side was judged to be difficult and risky to dissect owing to tumor location, poor visualization and difficulty in handling surgical devices. Therefore, TMA was sequentially performed, and the tumor was completely and safely resected. The intraoperative diagnosis was schwannoma. The patient was discharged 1 week after the operation. The final diagnosis was also schwannoma, and the tumor was completely resected. Horner syndrome appeared transiently, but he was well without Horner syndrome 1 year postoperatively. He had no functional restriction of the left upper extremity.

## Discussion

The surgical approach for mediastinal tumors in the thoracic outlet is difficult to decide [1]. Because most of these tumors are benign, minimally invasive surgery is desirable. For small tumors, resection can be performed with simple VATS or VATS with carbon dioxide insufflation into the thoracic cavity, or, in some cases, robotic surgery; however, when tumors are large and adjacent to major vessel or nerves, it is often difficult to dissect the cranial part safely because of anatomical issues, surgical device issues in VATS, and poor visualization. In these cases, an additional neck approach is necessary for safety.

TMA was introduced by Grunenwald and Spaggiari for resecting superior sulcus tumors [2]. It is a useful and safe approach because it allows good visualization of major vessels and nerves in the neck without altering shoulder mobility. Based on these advantages of TMA and VATS, there are some reports on the combined surgical technique with TMA and VATS in the resection of superior sulcus tumors [3, 4], and we applied this technique to large mediastinal tumors in thoracic outlet adjacent to major vessels. Because it was difficult to obtain a good visualization of the opposite side with either the VATS or TMA approach, we considered that the combination of the VATS and TMA approach would complement each other. Furthermore, this dual approach enables a safer and less invasive operation procedure, reducing the risks of functional restriction or paresthesia of the upper extremity.

Postoperatively, Horner syndrome was detected in both cases, which both resolved naturally. This probably developed because of sympathetic nerve paresis induced by the influence of dissection around the sympathetic nerve. By this dual approach, the good visualization of sympathetic nerve, phrenic nerve and vagal nerve was achieved, and the injury of these nerves was avoided.

## Conclusion

The dual approach with TMA and VATS allows a safe and less invasive resection of large mediastinal tumors in the thoracic outlet. Although the transient Horner syndrome was detected postoperatively, it was naturally recovered.

## Abbreviations

CT: Computed tomography
TMA: Transmanubrial osteomuscular sparing approach
VATS: Video-assisted thoracoscopic surgery

## References

[1] Yamaguchi M, Yoshino I, Kameyama T, Osoegawa A, Tagawa T, Maehara Y (2006). Thoracoscopic surgery combined with a supraclavicular approach for removing a cervico-mediastinal neurogenic tumor. Ann Thorac Cardiovasc Surg.

[2] Grunenwald D, Spaggiari L (1997). Transmanubrial osteomuscular sparing approach for apical chest tumors. Ann Thorac Surg.

[3] Yokoyama Y, Chen F, Aoyama A, Date H (2014). Combined operative technique with anterior surgical approach and video-assisted thoracoscopic surgical lobectomy for anterior superior sulcus tumors. Interact Cardiovasc Thorac Surg.

[4] Shikuma K, Miyahara R, Osako T (2012). Transmanubrial approach combined with video-assisted approach for superior sulcus tumors. Ann Thorac Surg.

